# Skeletal Age Evaluation with BAUSport^TM^ Ultrasound in Young Slovak Female Athletes and Non-Athletes—A Cross-Sectional Study

**DOI:** 10.3390/jfmk11020215

**Published:** 2026-05-28

**Authors:** Petra Švábová (nee Uhrová), Iveta Cihová, Michal Soták, Darina Falbová, Lenka Vorobeľová, Radoslav Beňuš, Lucia Bundová

**Affiliations:** 1 Department of Anthropology, Faculty of Natural Sciences, Comenius University Bratislava, Ilkovičova 6, Mlynská dolina, 842 15 Bratislava, Slovakia; sotak11@uniba.sk (M.S.); darina.falbova@uniba.sk (D.F.); lenka.vorobelova@uniba.sk (L.V.); 2 National Sports Centre, Olympijské námestie 14290/1, 831 04 Bratislava, Slovakia; iveta.cihova@uniba.sk (I.C.); lucia.bundova@uniba.sk (L.B.); 3 Department of Track and Field, Faculty of Physical Education and Sports, Comenius University Bratislava, Nábr. arm. gen. L. Svobodu 9, 814 69 Bratislava, Slovakia; 4 Department of Pre-Primary and Primary Education, Faculty of Education, Comenius University Bratislava, Šoltésovej 4, 811 08 Bratislava, Slovakia

**Keywords:** skeletal age, maturation timing, ultrasound, youth athletes, volleyball, BAUSport^TM^

## Abstract

**Background:** Biological maturity assessment is increasingly discussed in youth sport because chronological age alone does not reflect inter-individual variation in growth and pubertal timing. Ultrasound-based systems such as BAUSport™ provide a radiation-free alternative to radiographic skeletal age assessment. This exploratory cross-sectional study examined skeletal age (SA) in Slovak female volleyball players, track-and-field athletes, and non-athletes using BAUSport™, while acknowledging substantial age differences between groups. **Methods:** Eighty girls (55 non-athletes, 13 volleyball players, 12 track-and-field athletes; aged 6.85–14.41 years) underwent anthropometric measurement and skeletal age (SA) assessment with BAUSport™. Chronological age (CA) was calculated as decimal age from birth date to measurement date. Groups were not age-matched; volleyball players were on average markedly older than the other groups. Skeletal maturity was categorized as early, on-time, or late using a ±1.0-year SA–CA threshold. Menarche status was recorded as an indicator of pubertal development. Group differences were evaluated using chi-square or Fisher’s exact tests as appropriate, while differences in SA and SA–CA according to menarche status were assessed using Welch’s *t*-test. **Results:** Volleyball players were older (12.90 ± 0.95 years) than non-athletes (10.22 ± 2.40 years) and track-and-field athletes (8.47 ± 1.51 years). They also demonstrated the largest mean SA–CA difference (+1.98 ± 0.73 years). The distribution of maturity categories differed across groups (χ^2^(4) = 14.32, *p* = 0.006, Cramer’s V = 0.30). Post-menarcheal girls exhibited significantly higher skeletal age and SA–CA values compared to pre-menarcheal girls. However, the substantial age disparity between groups represents a primary confounding factor and limits interpretation of sport-specific effects. **Conclusions:** This study demonstrates the practical application of BAUSport™ for rapid, radiation-free skeletal age assessment in youth. Differences according to menarche status support the biological relevance of skeletal maturity assessment. However, no valid between-group sport-specific inferences can be made because the groups were not age-matched and puberty is the dominant driver of skeletal maturation. Larger, age-matched studies are required before drawing conclusions regarding sport-specific selection patterns.

## 1. Introduction

Growth and development in children represent complex biological processes influenced by genetic, hormonal, environmental, and lifestyle-related factors. While chronological age reflects the calendar time elapsed since birth, biological age provides a more informative indicator of physiological development and maturation status [1]. Biological age comprises several interrelated components—skeletal age, dental age, sexual maturation, proportional age, and growth age—each progressing at its own tempo and contributing to overall developmental variability. Among these, skeletal age (SA) is widely regarded as a key marker of biological maturity, particularly during late childhood and adolescence, when inter-individual differences in growth velocity and pubertal timing become pronounced [2].

Skeletal age has traditionally been assessed using radiographic imaging of the left hand and wrist according to standardized atlases, most commonly the Greulich–Pyle (GP) and Tanner–Whitehouse (TW) methods. Ossification centers of the hand and wrist appear and fuse at predictable developmental stages, forming the basis for these standardized assessments [3]. Although radiographic evaluation remains the reference method, repeated exposure to ionizing radiation limits its suitability for longitudinal monitoring in pediatric and sport settings [4,5]. To address this limitation, non-invasive ultrasound-based approaches have been increasingly explored in recent years [5,6,7]. Unlike radiographic techniques, ultrasound-based systems evaluate structural characteristics of ossification centers without radiation exposure. The BAUSport™ system (SonicBone Medical Ltd., Tel-Aviv, Israel) is an imaging-based ultrasound device that utilizes B-mode grayscale imaging of the distal radius, selected metacarpal epiphyses, and the proximal phalanx of the third finger. Ultrasound-based skeletal age assessment with the BAUSport™ system is based on quantitative ultrasound principles. The device measures the speed of propagation and attenuation of high-frequency ultrasound waves as they pass through bone and cartilaginous structures. These acoustic properties differ between cartilage and mineralized bone tissue and change progressively during ossification and epiphyseal fusion. The acquired signals are processed using proprietary algorithms based on the Tanner–Whitehouse method to estimate skeletal age. Previous investigations have reported high agreement between BAUSport™ and radiographic reference methods in pediatric and athletic populations [7,8]. Additional studies have demonstrated the broader applicability of ultrasound-based skeletal age assessment in developmental research contexts [9,10,11].

Initial validation demonstrated strong agreement with radiographic standards, showing that skeletal age estimates derived from BAUS were consistent with traditional radiographic assessments [12]. More recently, Cumming et al. [8] confirmed high levels of agreement between BAUSport™ and the Fels method, reporting intraclass correlations of r = 0.98 for skeletal age and r = 0.93 for the skeletal age–chronological age difference, together with substantial agreement in maturation timing classification. Further studies have compared BAUSport™ with non-radiographic approaches. Ruf et al. [13] examined its association with the Khamis–Roche method for predicting adult height and demonstrated comparable accuracy, supporting its applicability in growth and performance monitoring. Cular et al. [14] evaluated BAUSport™ against the Moore and Mirwald method for estimating the timing of peak height velocity. While the offset method remained a useful screening tool, BAUSport™ provided a more direct and biologically grounded assessment of skeletal maturation timing.

Overall, these findings indicate that BAUSport™ represents a valid and reliable method for assessing skeletal maturity, with mean absolute errors typically below one year. However, minor systematic biases relative to radiographic reference methods have been reported and should be considered when interpreting results [14]. Given that the BAUSport™ system has already been validated against established standards, the present study focused on its practical application and interpretation rather than revalidation.

Biological maturation is influenced by multiple intrinsic and extrinsic factors. Low birth weight and malnutrition are frequently associated with delayed skeletal development [15,16], whereas overweight and obesity may accelerate maturation tempo [17]. Chronic inflammatory diseases, endocrine disorders, and autoimmune conditions may disrupt physiological growth patterns and lead to discrepancies between skeletal and chronological age [18,19]. Conversely, regular weight-bearing physical activity has been associated with enhanced osteogenesis and may influence bone development during childhood and adolescence [20]. These interacting biological and environmental influences contribute to substantial heterogeneity in maturation status among individuals of the same chronological age. In girls, menarche represents a key milestone of pubertal development and is commonly used as a non-invasive indicator of maturation status. As skeletal maturation is closely linked to pubertal development [15,16,19], menarche status may provide a useful external reference point when interpreting skeletal age.

In addition to individual determinants, broader secular trends must be considered. Over the past century, populations have exhibited earlier pubertal onset and increased average body height compared with previous generations, likely reflecting improvements in nutrition, healthcare, and living conditions [21,22,23,24]. As a result, contemporary cohorts may present with skeletal age values that differ from historical reference standards, which has implications for both clinical interpretation and sport-related evaluation.

Within youth sport, biological maturation has received growing attention due to its potential influence on performance, training readiness, and selection processes. Modern athlete development models increasingly emphasize long-term monitoring rather than single-time talent identification [25,26]. Nevertheless, many selection systems continue to rely predominantly on chronological age and short-term performance metrics, often without sufficient consideration of maturity-related variation [27,28]. Because biological age reflects the tempo of growth and pubertal progression, it may provide complementary information when evaluating youth athletes [27,29,30].

The Relative Age Effect (RAE) further complicates this landscape by contributing to unequal representation of athletes born earlier within the selection year [31]. Relatively older athletes within the same chronological category may also exhibit more advanced biological maturation, thereby amplifying physical advantages. One proposed approach to mitigate such disparities is bio-banding, whereby athletes are grouped according to biological rather than chronological age [30,32,33]. However, the practical implementation of maturity-based grouping requires reliable and feasible assessment methods.

Previous research has reported that early-maturing girls are often overrepresented in certain competitive sport environments, potentially due to temporary advantages in stature, strength, and coordination [31,34,35]. At the same time, longitudinal evidence suggests that early maturational advantages do not necessarily translate into superior long-term performance outcomes, as later-maturing athletes may subsequently achieve comparable or greater success once maturation differences diminish [27,36]. These findings highlight the importance of distinguishing temporary maturational differences from stable performance potential.

Given these considerations, the present study was designed as a small exploratory cross-sectional investigation examining skeletal maturity in a Slovak youth sample using the BAUSport™ ultrasound system. The study included three groups: girls participating in organized volleyball training, girls participating in organized track-and-field training, and girls engaging only in recreational physical activity (non-athletes). Because the groups were not age-matched and differed substantially in mean chronological age, the analyses were descriptive and hypothesis-generating rather than confirmatory. Importantly, no valid between-group sport-specific inferences can be made, as the groups were not age-matched. Puberty is the dominant driver of skeletal maturation, with menarche representing a key indicator of pubertal status in girls. Accordingly, the primary aim was to describe skeletal maturity patterns within groups and across developmental stages, rather than to infer sport-specific effects. The distribution of early, on-time, and late maturation categories across groups was therefore evaluated in an exploratory manner, and the results should be interpreted descriptively.

## 2. Materials and Methods

### 2.1. Participants

A total of 80 girls of European ancestry from Slovakia were examined and measured, including 55 non-athlete females, 13 female volleyball players, and 12 female track-and-field athletes aged between 6.85 and 14.41 years. Chronological age (CA) was calculated as decimal age from birth date to measurement date. The study was conducted as a small exploratory cross-sectional investigation using a convenience sample. Importantly, the groups were not age-matched. Volleyball players were substantially older on average than both non-athletes and track-and-field athletes, a factor that was considered in the interpretation of the results. The study formed part of an ongoing research project on the young Slovak population and was approved by the Ethics Committee of the Faculty of Natural Sciences, Comenius University in Bratislava (11 October 2022; protocol code ECH19026). Participation was voluntary and based on written informed consent obtained from parents or legal guardians. All procedures complied with the Declaration of Helsinki. Participants were informed about study objectives, procedures, and the non-invasive nature of the assessment. The purpose of the study was clearly explained to participants and their legal guardians, and participation had no influence on sport selection, training processes, or talent identification decisions. Each participant was assigned a unique identification code to ensure anonymity. Data were stored securely and used exclusively for research purposes. Participants were informed of their right to withdraw at any time. No medical screening for endocrine/growth disorders was performed.

The study sample consisted of the following:

- 55 non-athlete girls, who, if they engaged in sports, did so only recreationally (less than twice per week), with chronological ages ranging from 6.92 to 14.41 years (mean = 10.22 ± 2.40 years).
- 13 female volleyball players, aged 11.31 to 13.86 years (mean = 12.90 ± 0.95 years).
- 12 female track-and-field athletes, aged 6.85 to 10.86 years (mean = 8.47 ± 1.51 years).

The volleyball players participated in organized training activities with a frequency of five sessions per week. The track-and-field athletes participated in training activities at a minimum frequency of two sessions per week.

Inclusion criteria were: female sex; age between 6 and 14.5 years; European ancestry; participation in organized volleyball training, organized track-and-field training, or recreational/non-organized physical activity; and informed consent from a parent or legal guardian. Exclusion criteria included: previous fracture or injury of the left hand or forearm that could affect ultrasound assessment; chronic musculoskeletal or growth-related disorders known to influence skeletal maturation; and inability to complete anthropometric or ultrasound measurements.

### 2.2. Procedures

#### 2.2.1. Anthropometry, Skeletal Maturation Timing, and Skeletal Age

Stature and body weight were recorded using standardized anthropometric instruments—a stadiometer (to the nearest 0.1 cm) and a calibrated digital scale (to the nearest 0.1 kg)—in accordance with conventional anthropometric protocols.

Information on menarche status (pre-/post-menarche) was obtained from participants or their legal guardians as part of the standard data collection procedure during BAUSport™ assessment and was used as an indicator of pubertal status.

**Figure 1:**
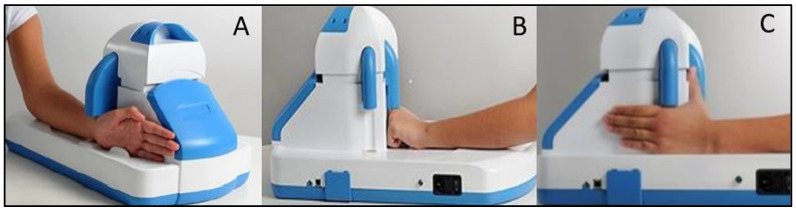
BAUSport^TM^ Sonic Bone protocol measurement—(**A**) 1st hand measurements position—wrist; (**B**) 2nd hand measurements position—phalanges; (**C**) 3rd hand measurements position—metacarpals (https://sonicbonemedical.com/product/, accessed on 20 February 2026).

**Figure 2:**
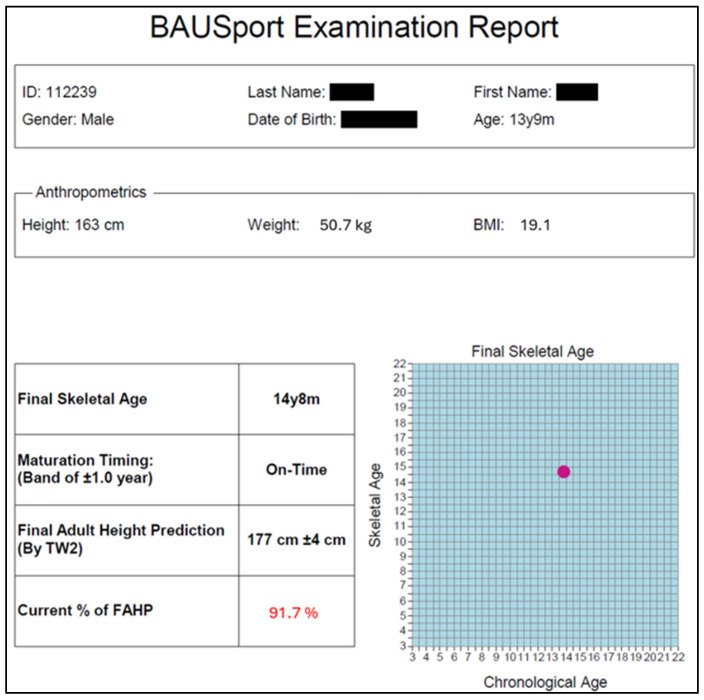
Illustration of derived skeletal age, maturation timing and final adult height prediction (FAHP) using the BAUSport™ Sonic Bone system; % FAHP indicates the proportion of predicted adult height already attained at the time of measurement.

Skeletal maturation timing was assessed using the non-invasive BAUSport™ ultrasound device (SonicBone Medical Ltd., Tel-Aviv, Israel), which evaluates three anatomical sites on the left hand—the distal radius/ulna, the distal epiphyses of the metacarpals, and the proximal shaft of the third phalanx (Figure 1). The system is based on quantitative ultrasound (QUS) principles, in which high-frequency ultrasound waves are transmitted through skeletal structures. It evaluates parameters such as the speed of sound (SOS) and ultrasound attenuation, reflecting the interaction of the acoustic signal with bone and cartilaginous tissues. These properties change during ossification and epiphyseal fusion and are used to estimate skeletal maturity [6,7,8]. After each scan acquisition, skeletal age (SA) was derived automatically by the manufacturer’s proprietary software (BAUS v. 1.0.0.12) using an algorithm based on the TW2 methodology (proprietary formula protected by nondisclosure agreement) together with predicted adult stature (Figure 2) [6,7,8].

Measurements were performed exclusively by one trained examiner (P.Š.).

Each measurement session required approximately 4 to 6 min. The software also provided an estimate of predicted adult height (based on the TW2 approach). Categories of maturation timing were defined by comparing SA to chronological age (CA):

- Late maturation timing: SA more than 1.0 year younger than CA.
- On-time maturation timing: SA within ±1.0 year of CA.
- Early maturation timing: SA more than 1.0 year older than CA.

The ±1.0-year threshold is commonly applied in youth sport and growth research [24,37] and approximates the standard deviation of skeletal age within one-year chronological age cohorts. However, it should be noted that the variability of SA–CA differences is not constant across development. The standard deviation is typically smaller in early childhood and larger during puberty; therefore, use of a fixed ±1.0-year threshold may increase the likelihood of classifying pubertal adolescents—such as the older volleyball players—as “early” or “late.” The resulting dataset was used to examine relationships between chronological age, skeletal age, predicted adult height, and maturity category distribution.

In the present study, only two outputs from the BAUSport™ system were used—skeletal age and skeletal maturation timing. Other available parameters (such as the prediction of adult height or anthropometric parameters—height, weight) were not included in the analyses, as the purpose of this preliminary work was solely to compare sport and non-sport groups in terms of skeletal age and maturation timing.

#### 2.2.2. The Accuracy and Validity of the SonicBone Device

The accuracy and validity of the BAUSport™ system have been evaluated in several independent studies. Initial validation demonstrated strong agreement with radiographic standards, showing that skeletal age estimates derived from BAUSport™ were consistent with traditional radiographic assessments [35]. More recently, Cumming et al. [9] confirmed high levels of agreement between BAUSport™ and the Fels method, reporting intraclass correlations of r = 0.98 for skeletal age and r = 0.93 for the skeletal age–chronological age difference, together with substantial agreement in maturation timing classification. These findings indicate that BAUSport™ provides reliable estimates of skeletal maturation timing while eliminating exposure to ionizing radiation. Further work has compared BAUSport™ with non-radiographic approaches. Ruf et al. [13] examined its association with the Khamis–Roche method for predicting adult height and demonstrated comparable accuracy, thereby supporting the applicability of BAUSport™ in growth and performance monitoring. More recently, Cular et al. [14] evaluated BAUSport™ against the Moore and Mirwald method, a widely used anthropometric approach for estimating the timing of peak height velocity. While the offset method remained a useful screening tool, BAUSport™ was shown to provide a significant advancement by offering a direct, individualized, and biologically grounded assessment of skeletal maturation timing. These studies suggest that BAUSport™ represents a valid and reliable method for assessing biological/skeletal age, with mean absolute errors typically below one year. Nonetheless, some investigations have reported minor fixed biases when compared with radiographic references, which should be considered when interpreting outcomes [14]. Given that the BAUSport™ system has already been validated against established radiographic standards, this study focused on its practical application and interpretation rather than revalidation.

### 2.3. Statistical Analysis

The distribution of skeletal maturity categories (early, on-time, late) across groups (non-athletes, volleyball players, track-and-field athletes) was examined using the chi-square test of independence (χ^2^). When expected cell counts were less than five, Fisher’s exact test was applied. Cramer’s V was calculated as a measure of effect size. When omnibus tests were significant, pairwise comparisons with Bonferroni correction were conducted. Differences in skeletal age (SA) and SA–CA between pre- and post-menarcheal girls were assessed using Welch’s *t*-test due to unequal variances. The Mann–Whitney U test was additionally used as a non-parametric confirmation. Effect sizes were calculated using Cohen’s d. No a priori power calculation was performed due to the exploratory nature of the study and the use of a convenience sample. Statistical significance was set at *p* < 0.05. Analyses were conducted using GraphPad Prism version 9.0 and Jamovi version 2.3.28.

## 3. Results

As shown in Table 1, volleyball players were on average older (12.90 ± 0.95 years) compared to non-athletes (10.22 ± 2.40 years) and track-and-field athletes (8.47 ± 1.51 years). They were also the tallest (170.69 ± 4.40 cm) and heaviest (55.29 ± 4.51 kg), with the highest BMI (19.00 ± 1.77 kg/m^2^). Skeletal age was most advanced in volleyball players (14.88 ± 1.01 years), reflected in the largest positive SA–CA difference (+1.98 ± 0.73 years). Non-athletes showed intermediate values (stature 142.53 ± 15.09 cm; SA–CA +0.71 ± 1.16 years), while track-and-field athletes were the youngest group with the lowest stature (133.50 ± 10.40 cm) and body mass (30.59 ± 8.12 kg), and had SA–CA close to zero (+0.84 ± 0.91 years). These descriptive comparisons must be interpreted in the context of the marked age differences between groups and therefore do not allow valid sport-specific comparisons.

Menarche status was available for all participants and is summarized in Table 2. In the total sample, 60 girls (75.0%) were pre-menarcheal and 20 girls (25.0%) were post-menarcheal. The mean age at menarche was 11.35 ± 0.94 years (range 10–13 years).

Differences in skeletal maturity according to menarche status are presented in Table 3. Post-menarcheal girls exhibited significantly higher skeletal age compared to pre-menarcheal girls (Welch’s t = −10.74, *p* < 0.001), with a very large effect size (Cohen’s d = 2.23). A similar difference was observed for SA–CA (Welch’s t = −3.26, *p* = 0.002; Cohen’s d = 0.78), indicating more advanced skeletal maturation relative to chronological age in post-menarcheal girls.

Based on the data presented in Table 4, the distribution of skeletal maturation timing categories (on-time, early, late) differed significantly across the three groups (χ^2^(4) = 14.32, *p* = 0.006, Cramer’s V = 0.30). Pairwise comparisons with Bonferroni correction indicated that volleyball players exhibited a significantly higher proportion of girls in early maturation timing category compared with non-athletes (χ^2^(2) = 14.16, *p* = 0.00084, Bonferroni-corrected *p* < 0.05). There were no significant differences between non-athletes and track-and-field athletes (*p* = 0.873, Bonferroni-corrected *p* = 1.00). The difference between volleyball players and track-and-field athletes did not reach statistical significance after Bonferroni correction (χ^2^(2) = 7.43, *p* = 0.024, Bonferroni-corrected *p* = 0.073), likely reflecting limited statistical power and the substantial chronological age disparity between these subgroups.

The effect size (Cramer’s V ≈ 0.30) suggests a moderate association between group membership (non-athletes, volleyball players, track-and-field athletes) and skeletal maturation timing.

**Figure 3:**
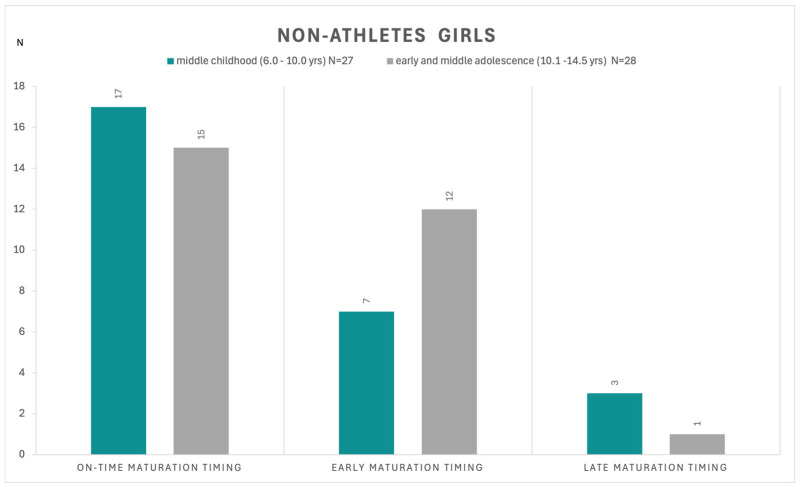
Skeletal maturation timing categories in non-athlete girls according to developmental stage (middle childhood vs. early and middle adolescence; *N* = 27 and *N* = 28).

When evaluating skeletal maturation timing in non-athlete girls, we stratified the sample according to developmental stage into middle childhood (6.0–10.0 years) and early and middle adolescence (10.1–14.5 years). In middle childhood (*N* = 27), 62.96% of girls were on-time in maturation timing, 25.93% were early in skeletal maturation timing, and 11.11% were late in maturation timing (Figure 3). In early and middle adolescence (*N* = 28), the proportion of on-time maturation timing decreased to 53.57%, while early maturation timing increased to 42.85%. Only 3.57% of girls in this age group were late in maturation timing.

**Figure 4:**
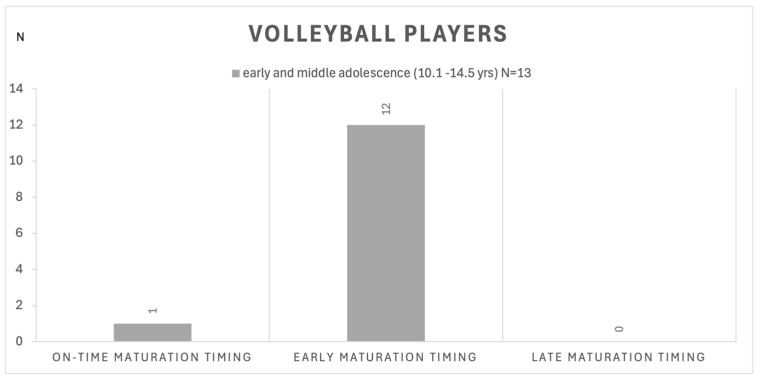
Skeletal maturation timing categories in adolescent volleyball players (early and middle adolescence; *N* = 13).

Among volleyball players (evaluated only in early and middle adolescence, *N* = 13), the majority (92.31%) was in the category of early maturation timing (Figure 4). On-time maturation timing was found in one girl (7.69%), whereas no cases of late maturation timing were recorded. Given their chronological age range (11–14 years) and corresponding anthropometric characteristics, many of the volleyball players were likely approaching skeletal maturity; maturity classification was based on the SA–CA criterion. Therefore, the classification based on maturation timing should be interpreted with caution. This pattern is consistent with their older age and more advanced developmental stage.

**Figure 5:**
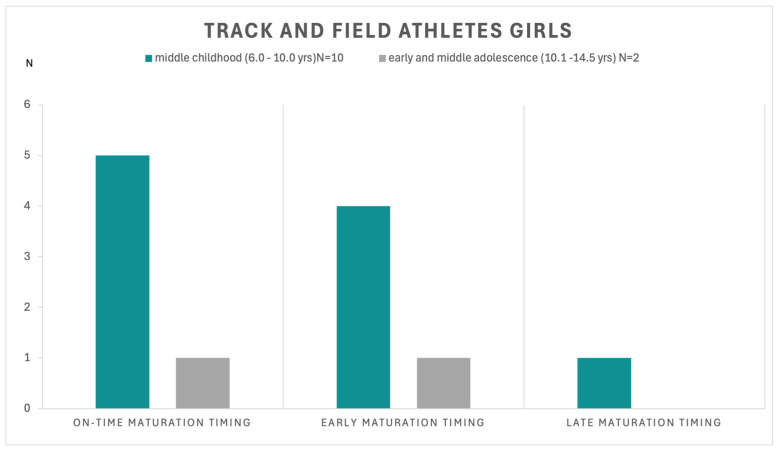
Skeletal maturation timing categories in track-and-field athletes according to developmental stage (middle childhood vs. early adolescence; *N* = 10 and *N* = 2).

In track-and-field athletes (Figure 5), also divided by developmental stage, the distribution was more balanced. In middle childhood (*N* = 10), 50% of girls were in the category of on-time maturation timing, 40% in early maturation timing, and 10% in late maturation timing. In early adolescence (*N* = 2), half of the girls were on-time and half early in maturation timing, with no late maturation timing observed. Given the small number of track-and-field athletes in early adolescence, stage-specific interpretation should be cautious.

These differences should be interpreted in the context of developmental stage differences rather than sport-specific effects, as the groups differ substantially in chronological age.

## 4. Discussion

A key finding of this study is the apparent difference in skeletal maturation patterns between groups. However, the most important factor underlying these differences is the substantial chronological age disparity between groups, as skeletal maturation is primarily driven by age and pubertal development. Early-maturation-timing individuals typically exhibit accelerated growth in height, body mass, and musculoskeletal development, which translates to improved strength and power capacities in adolescence [38]. Given their older age, the volleyball players in this study were likely at a more advanced stage of pubertal development, which is consistent with their higher skeletal age and greater prevalence of early maturation classification [39]. Maturity-related selection bias has been described in youth sport contexts, where athletes may be selected not only on skill, but also on maturational status and associated temporary physical advantages [40,41]. However, the most important limitation influencing interpretation of the present findings is the marked chronological age difference between groups. Volleyball players were on average approximately five years older than track-and-field athletes and approximately three years older than non-athletes. Puberty is one of the strongest drivers of skeletal maturation, and skeletal age is inherently associated with chronological age and pubertal stage [34]. Skeletal maturation is closely linked to pubertal development [15,16,19]. In girls, menarche represents a key milestone of pubertal development and serves as a practical indicator of maturation status. The strong differences observed between pre- and post-menarcheal girls in both skeletal age and SA–CA in the present study are therefore biologically consistent and further support the validity of skeletal age assessment. Consequently, the advanced skeletal age and the higher prevalence of “early” classification observed in volleyball players are very likely strongly confounded by their older age and more advanced developmental status. The current cross-sectional data do not allow separation of age-related maturation effects from potential sport-specific training exposure or selection processes. Therefore, the observed differences between groups cannot be interpreted as sport-specific effects but rather reflect differences in developmental stage.

Volleyball players in our sample exhibited a mean skeletal age advancement of +1.98 years relative to chronological age, with over 90% classified in the early maturation timing category. Similar patterns of advanced skeletal maturity have been reported in youth volleyball populations assessed using radiographic methods [41]. Likewise, Cular et al. [14] reported maturity advancement estimates in Croatian youth volleyball players using BAUSport™ compared with anthropometric maturity offset methods. While these comparisons are consistent with the possibility that volleyball cohorts may include a high proportion of biologically advanced athletes, the strong age imbalance in the current study means that sport-specific explanations must remain tentative.

It is therefore important to interpret the observed group differences primarily as a result of developmental stage differences, with sport-specific interpretations remaining speculative in the absence of age-matched comparisons. Skeletal age is closely associated with chronological age, stature, and BMI; thus, the observed differences may reflect normal age-related maturation patterns in addition to any selection mechanisms. Previous work [34] has shown that early-maturing girls are more likely to be selected into sports where body size provides performance advantages, including volleyball. The present results may be compatible with that literature, but they do not provide confirmatory evidence of selection bias because age and pubertal stage were not controlled and the design is cross-sectional.

In contrast, our study track-and-field athletes showed a more balanced distribution between on-time and early maturation timing individuals. However, this group was substantially younger and consisted primarily of girls in middle childhood. At younger ages, skeletal maturity variability is generally smaller than during adolescence, and the small number of track-and-field athletes in early adolescence further limits inference. Therefore, the present data do not allow conclusions regarding discipline-specific selection patterns in track-and-field athletes. Nevertheless, prior work suggests that maturity-related selection pressures may be less pronounced in athletics than in some team sports, depending on event demands and development pathways [42].

The division of the sample into two developmental stages (middle childhood vs. early adolescence) was applied to account for developmental differences in skeletal maturation, which become more pronounced during adolescence. This subdivision allowed descriptive evaluation of maturity patterns within broadly comparable developmental phases. The higher proportion of early maturers observed in early adolescence across groups is consistent with known developmental patterns and with evidence that maturity-related differences may become more salient during the pubertal transition [17].

The concept of accelerated growth and early maturation in some sport contexts has been observed across multiple disciplines. Kozieł et al. [43] reported earlier timing of peak height velocity in sports emphasizing power and speed, and Eichelberger et al. [23] discussed secular acceleration in biological maturation. Although the magnitude of SA–CA advancement in the volleyball group in the present study is within the range reported in some athletic samples, the age imbalance means that this pattern cannot be attributed to training exposure or selection without age-matched comparisons.

Among non-athlete girls, skeletal maturation timing categories were more balanced, with 61% classified as on-time and only 32% as early maturation timing girls. These proportions are consistent with data where early maturation prevalence is commonly reported in the range of approximately 25–35% [2,4], supporting that the non-athlete group broadly reflects expected population patterns.

From an applied perspective, biological maturity assessment remains a relevant consideration in youth sport development. Nevertheless, practical recommendations (e.g., routine maturity screening for talent identification or claims that BAUSport™ can identify late-maturing “high potential” athletes within Slovak volleyball) are not directly supported by the present cross-sectional, non-age-matched dataset. If future age-matched and longitudinal studies confirm discipline-specific maturity distributions and selection patterns, radiation-free tools such as BAUSport™ may prove valuable for growth monitoring and for informing developmentally appropriate training and competition structures, including bio-banding strategies. Overall, the findings of this study should be interpreted within a developmental framework, where age and pubertal status represent the primary determinants of skeletal maturation.

### Study Limits

Several important limitations must be acknowledged: Volleyball players were on average approximately 2.7 years older than non-athletes and approximately 4.4 years older than track-and-field athletes, placing them in a different maturational window. Volleyball players were therefore substantially older than both track-and-field athletes and non-athletes. Because puberty is a dominant driver of skeletal maturation, the apparent advancement in volleyball players is very likely strongly influenced by their older age and more advanced pubertal status. As a result, the groups are not directly comparable in chronological age, and sport-specific interpretations are limited. The data cannot determine whether sport participation influences maturation tempo or whether more biologically advanced individuals are preferentially selected into particular sports. No a priori power calculation was performed due to the exploratory nature of the study. The volleyball vs. track-and-field comparison in particular was likely underpowered given the small subgroup sizes. Inclusion of relatively older adolescents increases the likelihood that some participants approach skeletal maturity, which may affect classification and comparability across groups. Detailed pubertal staging (e.g., Tanner stage) was not assessed; however, menarche status was available and used as a proxy indicator of pubertal development. Participants were Slovak girls of European ancestry recruited from a single region, which limits generalizability to other populations. These limitations substantially restrict sport-specific conclusions and underscore the need for larger age-matched and longitudinal research.

## 5. Conclusions

This exploratory cross-sectional study demonstrates that BAUSport™ enables rapid, non-invasive, radiation-free skeletal age assessment in youth. Differences in skeletal maturity distribution were observed across groups. However, no valid between-group sport-specific inferences can be made, as the groups were not age-matched and puberty represents the dominant driver of skeletal maturation. The observed differences are therefore strongly influenced by marked chronological age disparities, particularly the older age of the volleyball group. The findings should be considered preliminary and descriptive. Differences according to menarche status further support the biological relevance of skeletal maturity assessment. Larger studies with age-matched groups and preferably longitudinal follow-up, incorporating direct pubertal assessment, are needed to determine whether maturity distributions differ systematically between sports and how skeletal maturity relates to long-term athlete development. If future controlled research confirms discipline-specific selection patterns, radiation-free ultrasound tools such as BAUSport™ may provide useful support for growth monitoring and for informing developmentally appropriate training and competition structures, including bio-banding.

## Figures and Tables

**Table 1:** Baseline characteristics of non-athletes, volleyball players, and track-and-field athletes.

	Non-Athletes (*N* = 55)				Volleyball Players (*N* = 13)				Track-and-Field Athletes (*N* = 12)			
Variable	Mean	SD	Min	Max	Mean	SD	Min	Max	Mean	SD	Min	Max
**CA (years)**	10.22	2.40	6.23	14.41	12.90	0.95	11.31	13.98	8.47	1.51	6.85	11.54
**Stature (cm)**	142.32	15.09	113.00	167.00	170.69	4.40	161.00	177.00	133.50	10.40	117.00	159.00
**Weight (kg)**	39.73	14.97	20.30	90.50	55.29	4.51	48.30	63.10	30.59	8.12	20.90	48.65
**BMI**	18.91	4.01	13.08	34.06	19.00	1.77	15.41	22.35	16.89	2.53	13.82	21.25
**SA (years)**	10.93	2.84	5.38	15.87	14.88	1.01	13.17	16.81	9.31	1.68	6.77	12.76
**SA–CA (years)**	0.71	1.16	−2.81	3.29	1.98	0.73	0.74	3.14	0.84	0.91	−1.00	2.32
**Predicted stature (cm) by Sonic Bone**	164.30	2.70	152.40	173.80	174.20	2.00	168.80	181.80	167.19	3.36	158.76	173.69

Legend: CA—chronological age, SA—skeletal age.

**Table 2:** Menarche status and age at menarche in the study sample.

Variable	Total Sample (*N* = 80)
Pre-menarche, *N* (%)	60 (75.0%)
Post-menarche, *N* (%)	20 (25.0%)
Age at menarche (years), mean ± SD	11.35 ± 0.94
Range (years)	10–13

Legend: Values are presented as counts and percentages or as mean ± standard deviation (SD).

**Table 3:** Skeletal age (SA) and SA–CA according to menarche status.

	Pre-Menarche (*N* = 60)	Post-Menarche (*N* = 20)	Welch’s t (df)	*p*-Value	Cohen’s d
SA (years)	10.23 ± 2.60	14.65 ± 1.06	−10.74 (≈70)	<0.001	2.23
SA–CA (years)	0.73 ± 1.18	1.55 ± 0.90	−3.26 (≈44)	0.002	0.78

Legend: Values are presented as mean ± standard deviation (SD). Welch’s *t*-test was used due to unequal variances. Cohen’s d represents effect size.

**Table 4:** Distribution of skeletal maturation timing categories among non-athletes, volleyball players, and track-and-field athletes.

	Non-Athletes (*N* = 55)		Volleyball Players (*N* = 13)		Track-and-Field Athletes (*N* = 12)	
	*N*	%	*N*	%	*N*	%
**On-time maturation timing**	32	58.18	1	7.69	6	50.00
**Early maturation timing**	19	34.55	12	92.31	5	41.67
**Late maturation timing**	4	7.27	0	0.00	1	8.33

## Data Availability

Data available on request due to restrictions (e.g., privacy, legal or ethical reasons). The data presented in this study are available on request from the corresponding author due to ethical reasons.

## References

[1] Yaneske E., Angione C. (2018). The poly-omics of ageing through individual-based metabolic modelling. BMC Bioinform..

[2] Satoh M. (2015). Bone age: Assessment methods and clinical applications. Clin. Pediatr. Endocrinol..

[3] Zhang A., Sayre J.W., Vachon L., Liu B.J., Huang H.K. (2009). Racial differences in growth patterns of children assessed on the basis of bone age. Radiology.

[4] Malina R.M. (2011). Skeletal age and age verification in youth sport. Sports Med..

[5] Rüeger E., Hutmacher N., Eichelberger P., Löcherbach C., Albrecht S., Romann M. (2022). Ultrasound imaging-based methods for assessing biological maturity during adolescence and possible application in youth sport: A scoping review. Children.

[6] Utczas K., Muzsnai A., Cameron N., Zsakai A., Bodzsar E.B. (2017). A comparison of skeletal maturity assessed by radiological and ultrasonic methods. Am. J. Hum. Biol..

[7] Wan J., Zhao Y., Feng Q., Sun Z., Zhang C. (2019). Potential value of conventional ultrasound in estimation of bone age in patients from birth to near adulthood. Ultrasound Med. Biol..

[8] Cumming S.P., Pi-Rusiñol R., Rodas G., Drobnic F., Rogol A.D. (2024). The validity of automatic methods for estimating skeletal age in young athletes: A comparison of the BAUSport ultrasound system and BoneXpert with the radiographic method of Fels. Biol. Sport.

[9] Kovács I., Kovács K., Gerván P., Utczás K., Oláh G., Tróznai Z., Berencsi A., Szakács H., Gombos F. (2022). Ultrasonic bone age fractionates cognitive abilities in adolescence. Sci. Rep..

[10] Ağırman K.T., Bilge O.M., Miloğlu Ö. (2018). Ultrasonography in determining pubertal growth and bone age. DMF Radiol..

[11] Brausch L., Dirksen R., Risser C., Schwab M., Stolz C., Tretbar S., Rohrer T., Hewener H. (2022). Classification of distal growth plate ossification states of the radius bone using a dedicated ultrasound device and machine learning techniques for bone age assessments. Appl. Sci..

[12] Rachmiel M., Naugolni L., Mazor-Aronovitch K., Koren-Morag N., Bistritzer T. (2017). Bone age assessments by quantitative ultrasound (sonicbone) and hand X-ray based methods are comparable. Isr. Med. Assoc. J..

[13] Ruf L., Cumming S., Härtel S., Hecksteden A., Drust B., Meyer T. (2021). Construct validity of age at predicted adult height and BAUS skeletal age to assess biological maturity in academy soccer. Ann. Hum. Biol..

[14] Cular D., Beslija T., Cavala M., Babic M., Kezic A. (2025). Comparative Analysis of Maturation Prediction Methods (Moore, Mirwald, BAUSport^TM^): Croatian Female Volleyball Youth Team Example. J. Funct. Morphol. Kinesiol..

[15] Martin D.D., Wit J.M., Hochberg Z., Sävendahl L., van Rijn R.R., Fricke O., Cameron N., Caliebe J., Hertel T., Kiepe D. (2011). The use of bone age in clinical practice—Part 1. Horm. Res. Paediatr..

[16] Martin D.D., Wit J.M., Hochberg Z., van Rijn R.R., Fricke O., Werther G., Cameron N., Hertel T., Wudy S.A., Butler G. (2011). The use of bone age in clinical practice—Part 2. Horm. Res. Paediatr..

[17] Zhou B., Qu X., Li M., Wang X., Xu Q., Wang J., Liu X., Zhang L., Zhang T., Gu J. (2015). Correlation of bone age development with overweight and obesity in 23,305 children from Beijing. Endocrine.

[18] Gupta N., Lustig R.H., Kohn M.A., Vittinghoff E. (2013). Determination of bone age in pediatric patients with Crohn’s disease should become part of routine care. Inflamm. Bowel Dis..

[19] Cavallo F., Mohn A., Chiarelli F., Giannini C. (2021). Evaluation of Bone Age in Children: A Mini-Review. Front. Pediatr..

[20] Zhang W., Wang X., Liu Y., He Q., Ding Q., Mei J., Li X. (2024). Effects of exercise on bone mass and bone metabolism in adolescents: A systematic review and meta-analysis. Front. Physiol..

[21] Wasiluk A., Saczuk J. (2025). Secular Trends in Height, Body Mass, and BMI Among Polish Boys in Eastern Regions from 1986 to 2021: Cross-Decade Analysis of Nutritional Status. J. Clin. Med..

[22] Kirchengast S., Juan A., Waldhoer T., Yang L. (2023). An increase in the developmental tempo affects the secular trend in height in male Austrian conscripts birth cohorts 1951–2002. Am. J. Hum. Biol..

[23] Eichelberger D.A., Chaouch A., Rousson V., Kakebeeke T.H., Caflisch J., Wehrle F.M., Jenni O.G. (2024). Secular trends in physical growth, biological maturation, and intelligence in children and adolescents born between 1978 and 1993. Front. Public Health.

[24] Pop R.-M., Tenenboum A., Pop M. (2021). Secular Trends in Height, Body Mass and Mean Menarche Age in Romanian Children and Adolescents, 1936–2016. Int. J. Environ. Res. Public Health.

[25] Shahidi S.H., Carlberg B., Kingsley J.D. (2023). Talent identification and development in youth sports: A systematic review. Int. J. Kinanthrop..

[26] Verbeek J., van der Steen S., Den Hartigh R.J.R., Van Yperen N.W. (2023). What do we currently know about the development of talent? A systematic review in the soccer context. Int. Rev. Sport Exerc. Psychol..

[27] Lloyd R.S., Oliver J.L., Faigenbaum A.D., Myer G.D., De Ste Croix M.B.A. (2014). Chronological age vs. biological maturation: Implications for exercise programming in youth. J. Strength Cond. Res..

[28] Wenger M., Csapo R. (2025). The relative age effect and the relationship between biological maturity and athletic performance in Austrian elite youth soccer players. Front. Sports Act. Living.

[29] Fransen J., Bennett K.J.M., Woods C.T., French-Collier N., Deprez D., Vaeyens R., Lenoir M. (2017). Modelling age-related changes in motor competence and physical fitness in high-level youth soccer players: Implications for talent identification and development. Sci. Med. Footb..

[30] Teixeira J.E., Forte P., Ferraz R., Monteiro A.M. (2022). Effects of chronological age, relative age, and biological maturation on accumulated training load and perceived exertion in youth football players. Front. Psychol..

[31] Cobley S., Baker J., Wattie N., McKenna J. (2009). Annual age-grouping and athlete development: A meta-analytical review of relative age effects in sport. Sports Med..

[32] Woods C.T., Raynor A.J., Bruce L., McDonald Z. (2016). Discriminating talent-identified junior Australian football players using a video decision-making task. J. Sports Sci..

[33] Baker J., Wilson S., Johnston K., Dehghansai N., Koenigsberg A., De Vegt S., Wattie N. (2020). Talent research in sport 1990–2018: A scoping review. Front. Psychol..

[34] Malina R.M., Rogol A.D., Cumming S.P., Coelho e Silva M.J., Figueiredo A.J. (2015). Biological maturation of youth athletes: Assessment and implications. Br. J. Sports Med..

[35] Romann M., Rössler R., Javet M., Faude O. (2017). Relative age effects in Swiss talent development: A nationwide analysis of all sports. J. Sports Sci..

[36] Vaeyens R., Lenoir M., Williams A.M., Philippaerts R.M. (2008). Talent identification and development programmes in sport: Current models and future directions. Sports Med..

[37] Malina R.M., Coelho-e-Silva M.J., Figueiredo A.J., Philippaerts R.M., Hirose N., Peña Reyes M.E., Gilli G., Benso A., Vaeyens R., Deprez D. (2018). Tanner–Whitehouse Skeletal Ages in Male Youth Soccer Players: TW2 or TW3?. Sports Med..

[38] Konarski J.M., Skrzypczak M., Freitas D., Malina R.M. (2024). Influence of body size and skeletal maturity status on strength and motor performances of soccer players 9–16 years. Sci. Rep..

[39] Albaladejo-Saura M., Vaquero-Cristóbal R., García-Roca J.A., Esparza-Ros F. (2022). The Effect of Age, Biological Maturation and Birth Quartile in the Kinanthropometric and Physical Fitness Differences between Male and Female Adolescent Volleyball Players. Children.

[40] Pinheiro J., Ribeiro L., Teixeira D., Ribeiro A., Coelho-E-Silva M.J. (2025). Skeletal Maturity in Adolescence: Evaluating Bone Development and Age Metrics. Diagnostics.

[41] de la Rubia A., Molina Martín J.J., Mon-López D., López-Serrano C. (2025). The Interactive Effect of Maturity Status and Relative Age on Physical Performance Within the Spanish Volleyball Federation’s Talent Pathway: Analysis by Sex and Playing Position. Sci.

[42] Bezuglov E., Shoshorina M., Emanov A., Semenyuk N., Shagiakhmetova L., Cherkashin A., Pirmakhanov B., Morgans R. (2022). The Relative Age Effect in the Best Track and Field Athletes Aged 10 to 15 Years Old. Sports.

[43] Kozieł S.M., Suder A., Chrzanowska M. (2024). Growth status and age at peak height velocity among youth participants in several sports: The Cracow longitudinal study. BMC Sports Sci. Med. Rehabil..

